# A Data-Driven Analysis of the Economic Cost of Non-Pharmaceutical Interventions: A Cross-Country Comparison of Kenya, Singapore, and Thailand

**DOI:** 10.3389/ijph.2022.1604854

**Published:** 2022-06-28

**Authors:** Jamaica Briones, Yi Wang, Juthamas Prawjaeng, Hwee Lin Wee, Angela Kairu, Stacey Orangi, Edwine Barasa, Yot Teerawattananon

**Affiliations:** ^1^ Saw Swee Hock School of Public Health, National University of Singapore, Singapore, Singapore; ^2^ Health Intervention and Technology Assessment Program, Ministry of Public Health, Nonthaburi, Thailand; ^3^ Health Economics Research Unit, KEMRI-Wellcome Trust Research Programme Nairobi, Nairobi, Kenya; ^4^ Centre for Tropical Medicine and Global Health, Nuffield Department of Medicine, Medical Sciences Division, University of Oxford, Oxford, United Kingdom

**Keywords:** COVID-19, non-pharmaceutical interventions, border closure, social distancing, economic impact

## Abstract

**Objective:** To estimate the economic impact of border closure and social distancing by estimating the decline of gross domestic product (GDP) in Kenya, Singapore and Thailand.

**Methods:** We analysed secondary data retrospectively. To calculate impact of NPIs on GDP, the relationship between GDP and stock market index was examined using ordinary least squares (OLS). Then, autoregressive and moving averages (ARMA) model was used to examine the impact of NPI on stock market index. The change in GDP due to NPIs was derived by multiplying coefficients of OLS and ARMA models.

**Results:** An increase in stock market index correlated with an increase in GDP, while both social distancing and border closure negatively correlated with stock market index. Implementation of NPIs correlated with the decline in GDP. Thai border closure had a greater decline in GDP than social distancing; Kenya exhibited the same trends; Singapore had the opposite trend.

**Conclusion:** We quantified the magnitude of economic impact of NPIs in terms of GDP decline by linking stock market index and GDP. This approach may be applicable in other settings.

## Introduction

While implementing non-pharmaceutical interventions (NPIs) for pandemic control has proven effective in reducing COVID-19 transmission, these interventions come with a high economic cost, especially those which involve movement restrictions such as social distancing and border closure [1]. According to the World Bank, global GDP dropped by 4.3% in 2020, which was largely attributed to the necessary halting of economic productivity [2]. As COVID-19 moves into an endemic status, governments will constantly have to calibrate reinstating or relaxing movement restriction measures, to attempt a balance between public health gains and economic losses [3, 4].

There is already considerable literature employing various time-series regression techniques to examine the relationship of NPIs with proxy economic indicators. One study demonstrated that high-frequency electricity market data can be used to estimate the causal, short-run impact of COVID-19 on the economy [5]. Other studies examined the effects of NPIs on stock market returns, albeit with conflicting results [1, 6–9]. Two studies reported that NPIs had a positive effect on stock market returns [7, 8], while two suggested that NPIs had a negative effect on stock market returns [6, 9]. It is worth noting that some of these studies suggest that government stimulus packages increase investor confidence, which reduces the negative economic effects of the pandemic [1, 9].

These existing studies, however, focused on stock market performance and had not directly quantified the economic losses caused by NPIs during COVID-19. Some studies which employed the human capital-based approach calculated the loss of productivity due to COVID-19, but did not consider that other economic players will adjust over time [10–12]. This study fills this gap by examining the correlation of GDP with the stock market index by using stock market index as an intermediate outcome to link effects of NPIs to GDP, using time-series regression techniques. We examined three countries with various economic development: Kenya, a lower-middle-income country; Singapore, a high-income country; Thailand, an upper-middle-income country.

This study contributes to the growing literature by providing quantitative insights into the effects of NPIs on the economy, using a novel method that can be applied in various settings. This approach can be a quick way to estimate the cost of NPIs, which can be used by policymakers to gauge the magnitude of the trade-off between economic losses and public health gains. Estimates from this paper may also be used as inputs in economic evaluations to identify optimal COVID-19 responses which can produce the greatest health and economic outcomes.

## Methods

**FIGURE 1:**
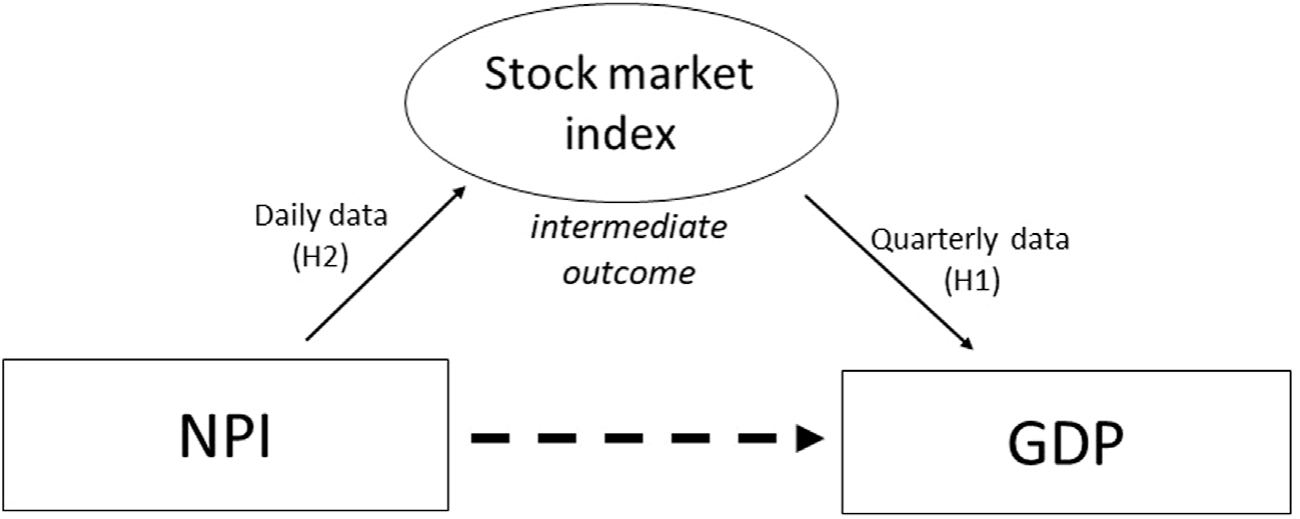
Linking NPI to GDP through stock market index (A data-driven analysis of the economic cost of Non-Pharmaceutical Interventions: A cross-country comparison of Kenya, Singapore and Thailand; Kenya, Singapore and Thailand; 2022).

Our main objective is to determine the effect of NPIs involving movement restrictions, particularly border closure and social distancing, on the GDP in three countries: Kenya, Singapore and Thailand. However ideal, estimating the effects of NPI through GDP directly is prohibitive due to the lack of granularity of data, given that GDP data are reported every quarter. We, therefore, hypothesized that stock market index—which has high-frequency data available—can serve as an intermediate outcome to infer the effects of NPIs on GDP (Figure 1).

Hypothesis 1 (H1) GDP and stock market index

We hypothesized a positive association between GDP and stock market index to determine whether stock market index can be a predictor of economic activity using quarterly data. We assume that stock market indices can reflect future expected economic outcomes, as they are made up of an aggregated measure of investment performance and can serve as a sentiment indicator that may reflect on the expected economy [1, 13–15].

Hypothesis 2 (H2) Stock market index and NPI introduction

Using high-frequency stock market data indices as dependent variables, we hypothesized that border closure and social distancing have a negative direct impact and positive indirect impact on the stock market [1]. For the direct effects, border closure and social distancing could have a negative effect on the stock market by adversely affecting economic activity. On the other hand, NPIs could have a positive indirect impact by weakening the negative market reaction to the growth in COVID-19 confirmed cases, if strict government actions reduce the intensity of local outbreaks. To highlight, the focus of this study is on the direct impact, through the reduction of economic activities.

This study originally intended to explore the effects of NPI on the sector-specific economy. However, given the limited publicly available data, we were only able to conduct such analysis for Thailand. Applying the same method as examining the overall GDP, we further examined the impact of NPI on sub-sector GDP to understand the validity of results using the sector-specific data. We hypothesize that industries such as tourism, transportation, consumer, construction, professional service, real estate, and finance will be affected more compared to industries such as health, agriculture, technology and electricity.

### Data

#### Gross Domestic Product

Data for GDP were retrieved from respective government websites of the three countries. Quarterly historical values were publicly available from 2000 to 2020 for Singapore and Thailand, while data from 2009 to 2020 were publicly available for Kenya. Real GDP (i.e., inflation-adjusted) was used in the analysis. Nominal GDP was multiplied to the GDP deflator for Singapore and Kenya to calculate the real GDP. For Thailand, the GDP chain dollar value was used since the GDP deflator was not available.

#### Stock Market Indices

Stock market indices used were Straits Times Index (STI), the market capitalisation weighted index of the top 30 companies listed on Singapore Exchange [16]; Stock Exchange of Thailand (SET) Index, comprising the prices of all common stocks on the main board of the SET for Thailand; and Nairobi Securities Exchange (NSE) 20, the weighted index of the top 20 companies listed on NSE for Kenya. These were selected since they were considered as the major stock market indices per country.

For H1, quarterly-average value of stock index was derived using the closing value of the stock market index. We chose the closing value for standardization since the stock market fluctuates significantly within a day. Meanwhile for H2, stock market index data closing values were also used and data were collected from Nov 2019 to Nov 2020. To identify the impact of the NPIs, dummy variables were generated (i.e., values as 0 before policy announcement, and 1 from day one of policy announcement). Announcement dates were used to consider anticipatory behaviours and were verified from official government websites [17–19].

#### Non-Pharmaceutical Intervention

We focused on NPIs related to movement restriction, specifically border closure and social distancing based on the Oxford COVID-19 Government Response Tracker (OxCGRT) [20]. Specifically, we aimed to understand the impacts of domestic movement restriction and international movement restriction. Given the difficulty to disentangle the effects of some NPIs since they were announced around the same time, i.e., multicollinearity issue, OxCGRT policy indicators that were similar in nature and implemented at the same time were combined and represented as a binary variable (Supplementary File S1). To classify the NPIs, we defined border closure as policy announcement of international movement restrictions, while social distancing as domestic movement restrictions. For domestic movement restrictions, we combined announcement of policies of (i) school closure, (ii) work closure, or (iii) restrictions in gathering. We further assumed that introducing and relaxing the NPIs have a symmetric effect on the GDP.

#### Confounders

Stock market indices are not only influenced by NPIs but also by other factors that can affect investors’ confidence and pandemic perception [1]. We included confounders to reflect local COVID-19 situation, other local policies besides the social distancing and border closure, global COVID-19 conditions and global economic conditions. Confounders listed below were considered in the H2 model, details of which can be found in Supplementary Table S1.

(a) COVID-19 related variables such as the daily number of global and local COVID-19 cases were used to reflect local COVID-19 situation [21]. NPIs might also have a positive economic impact with the reduction of the global and local COVID-19 cases since studies showed the growth in COVID-19 confirmed cases may be correlated with negative stock market returns [1, 22]. The indirect impact of these two confounders was incorporated using interaction terms in the Thailand model. A positive value for the estimated interaction term reflects the weakened negative market reaction to the growth in COVID-19 confirmed cases due to the NPIs.
(b) Weekly news trend using “coronavirus” term from Google trends was used as a gauge of public sentiment and panic due to the pandemic [23, 24]. These was considered as a continuous variable using Google search volume activity for the week [25].
(c) Dow Jones Index, oil and gold prices were used to control how the local stock market reacts to the global economic status [26]. These variables served as a measure of liquidity and stability which tend to deteriorate under shocks such as pandemics [6, 23]. These confounders were considered as continuous variables and were retrieved from Yahoo Finance.
(d) Availability of fiscal stimulus package were considered since investors may react positively to economic support programs since these programs may offset the negative effects of the NPIs [1, 9]. These were considered as binary variable for each respective country [1, 9, 20].

### Analytical Framework

Step 1 Estimating relationship between GDP and stock market index (H1)

**FIGURE 2:**
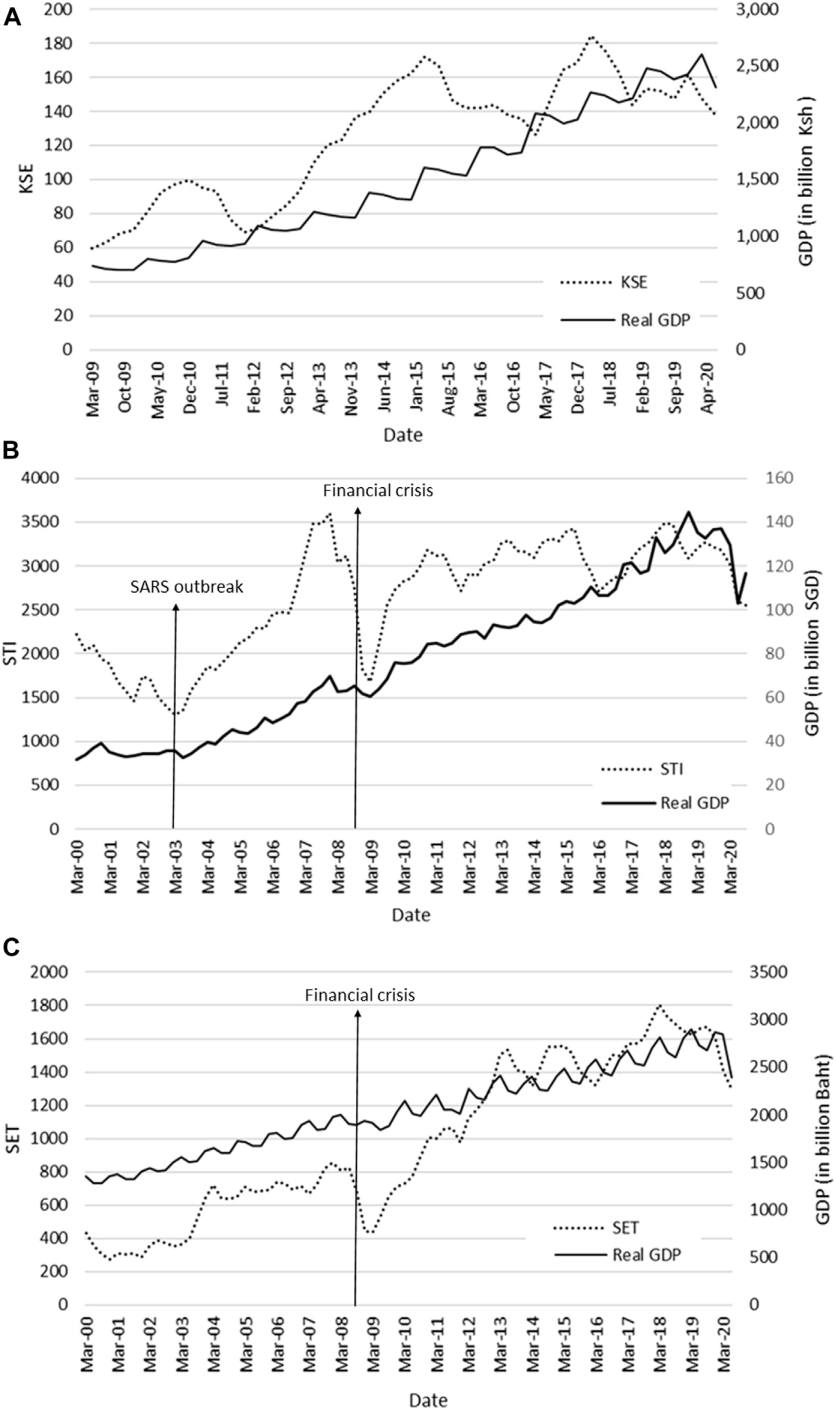
Quarterly GDP and stock market index plotted against time for Kenya **(A)**, Singapore **(B)**, and Thailand **(C)** (A data-driven analysis of the economic cost of Non-Pharmaceutical Interventions: A cross-country comparison of Kenya, Singapore and Thailand; Kenya, Singapore and Thailand; 2022).

Ordinary least squares (OLS) method was used to determine the relationship between GDP and stock market index (Figure 2). The regression model has the following form:

$$\text{d}1GDP_{t}=\beta_{0}+\beta_{1}*d1stocks_{t-1}+\beta_{2}*Q2+\beta_{3}*Q3+\beta_{4}*Q4+\varepsilon_{t}$$ (1)

where GDP refers to real GDP to adjust for inflation, and stocks refers to the mean stock market index for the lagged quarter; Q2 to Q4 are dummy variables indicating quarters within a calendar year (Q1 as an index category), and d1 indicates first difference.

For both GDP and stock market index, first difference was applied, e.g., $\text{d}1GDP_{t}=\;GDP_{t}-\;GDP_{t-1}$ , to transform the data into stationary. We performed Dickey-Fuller test to test for stationarity of data (Supplementary Table S2).

Step 2 Estimating the relationship between NPIs and stock market index (H2)

For H2, we examined the impact of social distancing and border closure on the stock market index using daily data. The analysis resembles a before and after comparison, taking also into consideration the time-series nature of the stock market index by using an Autoregressive Integrated Moving Average (ARMA) model (Eq. 2):

$$stocks_{t}=\mu_{0}+\mu_{1}*socialdis_{t}+\mu_{2}*borderclose_{t}+\gamma *confounders_{t}+\epsilon_{t}$$ (2)

where $socialdis_{t}$ is a dummy variable with value 1 when social distancing was implemented, and value 0 otherwise. $borderclose_{t}$ is a dummy variable with value 1 when border closure was implemented, and value 0 otherwise. $confounders_{t}$ is a vector consisting of all the confounders, while γ is a vector of coefficients. Finally, $\epsilon_{t}$ represents the random disturbances denoted through a polynomial of autoregressive (AR) order p and moving average (MA) order q for the ARMA (p, q) process as shown in (Eq. 3):

$$\epsilon_{t}=\;\overset{p}{\underset{j=1}{\sum }}\rho_{j}\epsilon_{t-j}+\;\overset{q}{\underset{l=1}{\sum }}\theta_{l}\phi_{t-l}+\phi_{t}$$ (3)

where $\epsilon_{t-j}$ is the lagged value of $\epsilon_{t}$ by a period j. $\rho_{j}$ is the autocorrelation parameter for $\epsilon_{t-j}$ . $\phi_{t-l}$ is the lagged value of $\phi_{t}$ by a period of l. $\phi_{t-q}$ to $\phi_{t}$ are independently distributed white noise following $N(0,\;\sigma^{2}$ ). $\theta_{l}$ is the moving-average parameter for $\phi_{t-l}$ . Akaike information criterion (AIC) and Bayesian information criterion (BIC) were used to determine the order of ARMA process, i.e.: values of p and q.

Results using stock market index in log-form were presented for the main analysis. Meanwhile, robustness tests through (i) use of original scale values; (ii) test for day of the week effect; (iii) use of ‘coronavirus vaccine’ term instead of ‘coronavirus’ term for news trend are shown at Supplementary Tables S3–S9.

Step 3 Estimating the change in GDP due to NPIs

The magnitude of change to the GDP was represented through the percentage reduction to GDP after announcement of each of the NPIs. Three methods were used to calculate the impact of NPI on GDP. Results using GDP and stock market index in log-scale were presented in the main text, while the methods and results using two methods in the original scale were presented in the Supplementary File. To estimate the change in GDP due to NPIs, we estimated the correlation between real GDP and stock market index, which is β1 from Step 1. From Step 2, we estimated the impact of NPIs on stock market index, which are $\mu_{1}$ and $\mu_{2}$ .

The impact of NPIs on GDP was estimated by multiplying the coefficients from Steps 1, 2. To account for uncertainties, five models were selected based on the results of AIC and BIC, which are AR (0); AR (1); AR (2); AR (1) MA (1); AR (1) MA (2). We presented the average effect of the NPIs along with the highest and lowest values resulting from the five models selected.

## Results

An increase in stock market index correlated with an increase in GDP across the countries examined for H1. The coefficient values derived were 0.066 (95% CI: −0.0093–0.142) for Kenya, 0.126 (95% CI: 0.00926–0.243) for Singapore and 0.083 (95% CI: 0.0298–0.136) for Thailand. Although only Singapore and Thailand resulted in a significant value (*p* < 0.05), we observed that the magnitude of correlation increased depending on the level of economic development of the country. In terms of the time-series graph, there is a general uptrend with the GDP in relation to stock market indices for the countries explored (Figure 2).

**FIGURE 3:**
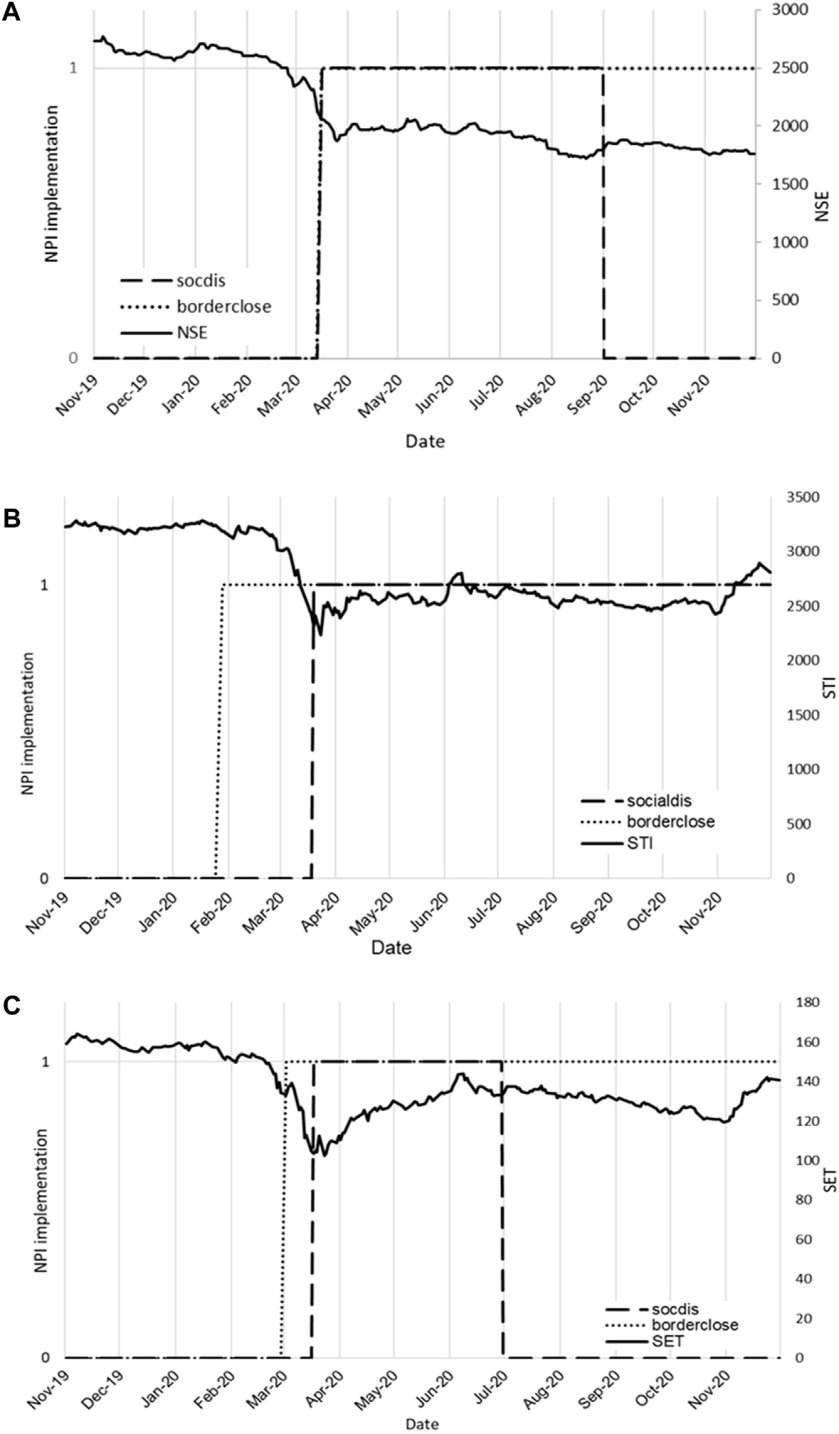
Daily stock market index plotted with timeline of NPI implementation (1: NPI present, 0: NPI absent) for Kenya **(A)**, Singapore **(B)** and Thailand **(C)** (A data-driven analysis of the economic cost of Non-Pharmaceutical Interventions: A cross-country comparison of Kenya, Singapore and Thailand; Kenya, Singapore and Thailand; 2022).

Meanwhile, regression results of H2 models showed that both social distancing and border closure negatively correlated with stock market index (Table 1). While the effects were not all statistically significant, there is a general pattern of decreasing stock market index when the NPIs were announced. The time-series graphs show the timeline of NPI announcement along with stock market index (Figure 3). As illustrated in the figure, there was a sharp decline in stock market index when the NPIs were announced [27]. To note, border closure was not lifted in the three countries during the period of analysis. On the other hand, social distancing was only implemented for specific periods for Thailand (March—July 2020) and Kenya (March 2020—September 2020), while social distancing was not lifted in Singapore since implementation from March 2020.

**TABLE 1:** Resulting coefficients for H2 model (A data-driven analysis of the economic cost of Non-Pharmaceutical Interventions: A cross-country comparison of Kenya, Singapore and Thailand, Singapore and Thailand; 2022).

	Models used in the analysis				
	AR (0)	AR (1)	AR (2)	AR (1) MA (1)	AR (1) MA (2)
Border closure					
Kenya	−0.118*	−0.0121	−0.0083	−0.0106	−0.0119
	(−0.156 to −0.0803)	(−0.0364 to 0.0122)	(−0.0338 to 0.0172)	(−0.0359 to 0.0148)	(−0.0393 to 0.0156)
Singapore	−0.0173*	−0.0141	−0.00141	−0.00596	−0.00269
	(−0.0342 to −0.000373)	(−0.0515 to 0.0232)	(−0.0196 to 0.0168)	(−0.0260 to 0.0141)	(−0.0248 to 0.0194)
Thailand	−0.0669*	−0.0371*	−0.0302*	−0.0278	−0.0309
	(−0.101 to −0.0332)	(−0.0811 to 0.00699)	(−0.0586 to −0.00174)	(−0.0582 to 0.00253)	(−0.0629 to 0.00114)
Social distancing					
Kenya	−0.0225*	−0.00722*	−0.00425	−0.00423	−0.00430
	(−0.0388 to −0.00607)	(−0.00906 to −0.00537)	(−0.0126 to 0.00413)	(−0.0136 to 0.00510)	(−0.0135 to 0.00488)
Singapore	−0.0902*	−0.107*	−0.0643*	−0.0718*	−0.0653*
	(−0.106 to −0.0744]	(−0.154 to −0.0590]	(−0.105 to −0.0239]	(−0.120 to −0.0239]	(−0.111 to −0.0198]
Thailand	−0.053*	−0.0199*	−0.0175*	−0.0192*	−0.0193*
	(−0.127 to 0.0207)	(−0.0379 to −0.00197)	(−0.0441 to 0.00900)	(−0.0457 to 0.00735)	(−0.0462 to 0.00764)

Note: *means significant values with *p* value <0.05, 95% confidence intervals in parenthesis.

For the final results, we observed an evident pattern of GDP decrease upon NPI announcement in the three countries examined (Table 2). In Thailand, a higher GDP decline was correlated with border closure (0.23%–0.56%), compared with social distancing (0.15%–0.44%). This pattern was similarly found in Kenya, with a higher GDP decline correlated with border closure (0.05%–0.785%), compared with social distancing (0.03%–0.15%). However in Singapore, a greater GDP decline was correlated with social distancing (0.81%–1.35%), compared with border closure (0.02%–0.22%).

**TABLE 2:** Estimated GDP reduction for border closure and social distancing, in percentage (A data−driven analysis of the economic cost of Non−Pharmaceutical Interventions: A cross-country comparison of Kenya, Singapore and Thailand; Kenya, Singapore and Thailand; 2022).

Country	Border closure			Social distancing		
	Best fit	Average	Range	Best fit	Average	Range
Kenya	−0.05	−0.21	−0.05 to −0.78	−0.03	−0.05	−0.03 to −0.15
Singapore	−0.02	−0.07	−0.02 to −0.22	−0.81	−0.94	−0.81 to −1.35
Thailand	−0.25	−0.30	−0.23 to −0.56	−0.15	−0.21	−0.15 to −0.44

Note: Best fit are not the same across three countries. Both the average and range (highest to lowest results) were derived from the five models.

For sector-specific results in Thailand, sectors such as tourism, professional services, consumer, transportation, real estate, and construction were found negatively correlated with both border closures and social distancing (Table 3). As expected, labour-intensive and domestically-oriented sectors such as construction, consumer, professional services, and real estate, as well as outward-oriented sectors such as tourism and transportation had a greater decline in GDP caused by the movement restrictions [28, 29]. On the other hand, sectors such as agricultural, technology, and health were seen unaffected by the announcement of NPIs. This may be the case since these sectors were less exposed to movement restrictions, and had outputs driven by domestic demand [29, 30].

**TABLE 3:** Estimated Sector GDP reduction for border closure and social distancing for Thailand, in percentage (A data-driven analysis of the economic cost of Non-Pharmaceutical Interventions: A cross-country comparison of Kenya, Singapore and Thailand; Kenya, Singapore and Thailand; 2022).

	Border closure			Social distancing		
	Best fit	Average	Range	Best fit	Average	Range
Tourism	−0.304	−0.492	−0.255 to −1.381	−1.043	−1.024	−0.823 to −1.085
Professional service	−0.672	−0.636	−0.514 to −0.672	−0.254	−0.287	−0.242 to −0.441
Consumer	−0.190	−0.380	−0.187 to −1.148	−0.113	−0.269	−0.102 to −0.893
Transportation	−0.256	−0.277	−0.228 to −0.427	−0.260	−0.147	0.335 to −0.272
Real estate	−0.030	−0.031	−0.028 to −0.034	−0.025	−0.015	0.020 to −0.026
Construction	0.128	0.175	0.374 to 0.121	−0.002	−0.002	0.002 to −0.003
Health	0.057	0.035	0.070 to −0.043	0.014	−0.004	0.002 to −0.003
Technology	−0.045	−0.057	−0.044 to −0.105	0.007	0.012	0.033 to 0.005
Electricity	−0.116	−0.146	−0.116 to −0.262	−0.013	0.024	0.180 to −0.021
Finance	0.035	0.036	0.038 to 0.034	0.042	0.031	0.042 to −0.008
Agriculture	0.204	0.154	0.205 to −0.043	0.098	0.274	0.985 to 0.088

Note: Best fit are not the same across three countries. Both the average and range (highest to lowest results) were derived from the five models.

## Discussion

How do we balance the need to control the pandemic with the need to sustain the economy? In this paper, we addressed this policy question indirectly by estimating the economic consequences of NPIs so that policymakers may reflect on their trade-offs, particularly for countries that are pursuing a zero COVID-19 policy. Data from three countries with different cultures, socioeconomic structures, and income levels were examined. We focused on two NPI categories involving movement restrictions—internal restrictions for social distancing and external restrictions for border closure.

We observed that the two NPI strategies differ in impact depending on the country. Specifically, border closure contributed to a greater GDP decline than social distancing for both Thailand and Kenya, while the opposite was observed in Singapore. The differences may be due to the level of strictness of the NPIs depending on the country. However, most governments had implemented strict containment policies on the first year of the pandemic as a general response [31, 32]. Countries should select NPIs that have a minimal impact on the economy as a way to contextualize the implementation of NPIs based on relevant economic considerations [32, 33].

Quite striking perhaps in our results was the low GDP reduction due to NPIs for all the countries explored. In 2020, the actual GDP contraction of Singapore was 5.8%, while Thailand’s GDP fell by 6.1%, and Kenya’s stood at 0.3% [34]. However small, this is aligned with the results of a study in Denmark and Sweden, where they suggest that social distancing laws cause only small losses of economic activity during the COVID-19 pandemic, and suggest that most of the GDP contraction is due to the virus itself [27]. Further, poor compliance with the mandated policies could also contribute to the small economic impact [35].

The difference in the impact of NPI on economic cost may be explained by the unique economic landscape of the country, as well as other NPIs implemented during the pandemic. The outward-oriented economies of Thailand may be the reason why the external-induced shock had a greater effect than the internal-induced shock, due to their dependence on sectors with heavy external demands such as tourism and transportation [36]. As a global trade and business hub, we expected Singapore would follow the same trend, however, our findings showed otherwise. A possible reason is that businesses in Singapore pivoted rapidly to digitalization and introduced products and services that suited pandemic times [37, 38]. In contrast, Kenya and Thailand heavily depend on manufacturing and agriculture, which are both highly vulnerable to disruptions in supply chains, and are more difficult to digitalize [39, 40].

We provided an opportunity to demonstrate the relationship between changes in actual GDP and specific NPI as an advantage to our statistical approach. A reduced-form analysis, using regression analysis guided by economic theory, was used to examine the impact of NPI on GPD. This served as a simple framework to calculate the cost of a particular or group of NPIs that can be easily applied to other settings. While using stock market index may arguably add some noise, most macroeconomic data are only available at low frequency making it difficult to predict developments early and reliably. For our first hypothesis, we examined the correlation between GDP and stock market index to demonstrate the reliability of using stock market index as an intermediate variable. Additionally, relevant confounders were added in the second hypothesis to address biases [41].

We explored several methods and robustness analyses to understand the magnitude of the impacts of NPIs on GDP, since it is difficult to ascertain whether upper or lower bounds were estimated. It is understood that not all aspects of the economy (GDP) are reflected by stock market indices. It may be the case in Kenya, where the stock market is less developed and an underestimation may occur. On the other hand, the economy corrects itself over time and people adjust. The cost of NPIs could decrease over time, which may result in an overestimation if we consider mid-term or long-term impact. Furthermore, other factors that impact economic growth such as natural and human resources, and technological advancement were also not incorporated in the model. These factors are important to consider if a longer time horizon was considered. Recognizing the complex context, we presented our main results in a range, which gives an estimate of the magnitude of the cost of NPIs.

### Limitations

One challenge was multicollinearity issues, with two NPIs being implemented almost simultaneously across the countries evaluated [7]. In general, we would expect that multicollinearity will lead to larger standard errors but would generate unbiased and consistent point estimates. For the case of Kenya, although the two NPIs start precisely at the same time, the date of relaxing the policy was different. Hence, we can still separately estimate the impacts of two NPIs on stock market index. An additional assumption required is that introducing and relaxing the NPI have a symmetric effect on GDP. Still, the proposed approach should be considered carefully depending on the implementation dates of the NPIs per country to determine the possibility of disentangling or further bundling them together. Further, we tried to address multicollinearity by combining related variables that are similar and are implemented around the same time, i.e., combining school closure, workplace closure, and restriction in gathering into domestic movement restriction [31]. While it is useful to further investigate the cost due to each policy, this is not feasible in the current study. In particular for school closure, there could be long-term cost due to a disruption of normal schooling as this may affect children’s productivity when they turning to adults.

Given the limitations of our data, our results must be viewed with caution as we acknowledge that our adjustments may not be adequate. The relationship between GDP and stock market index remains contentious and complicated, with all the exogenous variables [15]. Other important factors contribute to the GDP that are not captured with stock market index we used which can be an added reason for a low magnitude of impact for NPIs in our results. Different players and factors may move in different directions. Hence, we also explored the subsector stock market index but only for Thailand due to data availability. In terms of disruptive behaviour and economic stresses, for example, numerous players in the economic system such as households, private companies, and governments respond in various ways to NPIs [42]. Other important factors to consider are simultaneous public panic, and aversive travel behaviour [42].

Adding to the limitation of using stock market index as an intermediate variable, stock market index is more volatile in the short run than the GDP. In ideal circumstances, an indicator that can address these issues should be used, but due to the lack of granularity in GDP data, we chose stock market index instead. Furthermore, the relationship between GDP and stock market index changed in response to the current pandemic. Economic players would respond to the pandemic as people voluntarily reduce their social activities [27]. Hence, the long-term impact might be less pronounced compared to the short-run impact.

In this work, we do not claim our results are causal impact, as causal impact will require randomized control trial which is not possible for this study, or require econometric identification strategies which may not be easily replicable in another setting. Given the complicated nature of the problem, we did not attempt to incorporate these into the model as it may take years to build a model that fully captures the interaction of the various players in the economy. Our analysis is a reduce-form analysis aiming to overcome the data issue, i.e., lack of granularity of GDP data, by using stock market index as an intermediate variable. This study serves as a stepping stone towards these more complex studies.

However, it does not mean that we should not attempt to measure these, but we have to be far more cautious on how we interpret the results [43]. We aim to provide a reasonable estimate or at best, a measure of the magnitude of effect of a particular set of or all NPIs on the economy. The method can help policymakers understand the associated cost and make more informed decisions in response to a public health emergency. This work illustrates why it is prudent to constantly re-assess and re-evaluate policies to build a suitable strategy and prevent curtailment of living standards for too long.

### Conclusion

This study presents a novel approach by using stock market index as a proxy for economic activity and as an intermediate outcome to link NPIs with GDP change. By employing time series regression techniques, we illustrate that NPIs correlates to GDP decline. Depending on the country, the magnitude of effect of the two NPIs examined differ accordingly. Specifically, it was observed that border closure is correlated with higher magnitude of economic downturn compared with social distancing in Thailand and Kenya, and the opposite was seen in Singapore.

To our knowledge, this is the first paper to correlate the effects of NPIs to GDP change. Future research avenues may consider re-evaluating NPI consequences at this present time when COVID-19 vaccines are already administered. Furthermore, examining economic response to NPIs in other country settings would offer insights for further analysis.
