# Supplementary material for: A Data-Driven Analysis of the Economic Cost of Non-Pharmaceutical Interventions: A Cross-Country Comparison of Kenya, Singapore, and Thailand
Source: Int J Public Health. 2022 Jun 28;67:1604854. doi: 10.3389/ijph.2022.1604854 (PMC9273740; doi:10.3389/ijph.2022.1604854)
Supplement: Supplementary file 1 [file DataSheet1.docx]

**Supplementary File**

**Table S1.** Definition of all the variables used in the analysis (A data-driven analysis of the economic cost of Non-Pharmaceutical Interventions: A cross-country comparison of Kenya, Singapore and Thailand; Kenya, Singapore and Thailand; 2022)

| **Variable** | **What it measures** | **Data definition** | **Type of data** | **Available data frequency** | **Reference** |
| --- | --- | --- | --- | --- | --- |
| **Hypothesis 1** | | | | | |
| Real GDP | Overall economic activity | GDP 2000-2020 3^rd^ QTR, quarterly | Continuous | quarterly | Gov't websites |
| Stock market index | Proxy for economic activity | Average of the daily stock market index closing value, quarterly | Continuous | daily | Yahoo finance |
| **Hypothesis 2** | | | | | |
| Stock market index | Proxy for economic activity | Daily stock market index closing value | Continuous | Daily | Yahoo finance |
| Social distancing | Announcement of school or work closing, or restrictions in gathering | 0 - No measure in place | Binary | Daily | Oxford index, primary data |
|  |  | 1 - At least one of social distancing measures in place |  |  |  |
| Border closure | Announcement on restrictions on international travel Note: this records policy for foreign travellers, not citizens | 0 - Screening from some or all regions (temperature screening) or quarantine arrivals | Binary | Daily | Oxford index, primary data |
|  |  | 1 - Ban arrivals (healthy or sick) from some or all regions/ total border closure |  |  |  |
| Fiscal stimulus | Announcement of economic stimulus spending (either debt relief/income Note: *only record amount additional to previously announced spending* | 0 - No stimulus announcement | Binary | Daily | Oxford index, primary data |
|  |  | 1- Announcement of economic stimulus to mitigate the impact of the COVID-19 outbreak |  |  |  |
| Local COVID-19 cases | Fear/ sentiment | Reported daily COVID-19 cases | Continuous | Daily | Our World in Data |
| World COVID-19 cases | Sentiment | Reported daily COVID-19 cases in the world | Continuous | Daily | Our World in Data |
| Virus trend | Public sentiment | Interest over time with the topic *“coronavirus”* - Numbers represent search interest relative to the highest point on the chart for the given region and time. | Continuous | Weekly | Google trends |
| Gold price | Volatility | Gold is a hedge against political unrest and inflation. A portfolio allocation in commodities is done to lower overall portfolio risk. (explain why you need to account for volatility) | Continuous | Daily | Yahoo finance |
| Oil price | Volatility | Crude oil is an essential commodity that provides energy and petroleum products to the global market. Usually sensitive to inventory levels, production, global demand, interest rate policies, and aggregate economic figures such as gross domestic product. | Continuous | Daily | Yahoo finance |
| Dow Jones Index | US stock market | A stock market index that measures the stock performance of 30 large companies listed on stock exchanges in the United States. | Continuous | Daily | Yahoo finance |

We present the time-series graph for the news trend using ‘coronavirus’ search term in Singapore to show the upticks in


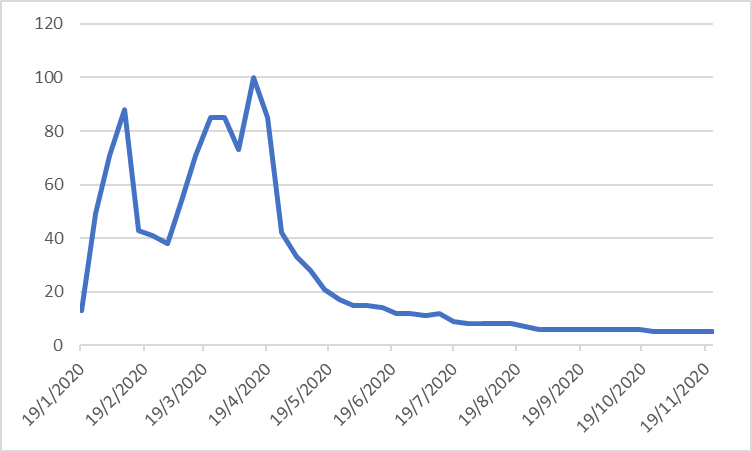


Figure S1. Time-series graph for the weekly news trend for ‘coronavirus’ search term in Singapore (A data-driven analysis of the economic cost of Non-Pharmaceutical Interventions: A cross-country comparison of Kenya, Singapore and Thailand; Kenya, Singapore and Thailand; 2022)

The table below presents the derived MacKinnon approximate p-value for Z(t) for each of the variable generated for H1. P-values < 0.05, means that the null hypothesis is rejected and that a unit root is present in the time series sample.

**Table S2. Dickey-Fuller test for unit root** (A data-driven analysis of the economic cost of Non-Pharmaceutical Interventions: A cross-country comparison of Kenya, Singapore and Thailand; Kenya, Singapore and Thailand; 2022)

| **Variables** | **P-values** |
| --- | --- |
| **Kenya** |  |
| lagNSE | 0.4282 |
| laglnNSE | 0.2367 |
| d1lagNSE | 0.0001 |
| d1laglnNSE | 0.0010 |
| GDPreal | 0.8735 |
| lnGDPreal | 0.7737 |
| d1GDPreal | 0.0000 |
| d1lnGDPreal | 0.0000 |
| **Singapore** | |
| lagSTI | 0.5238 |
| laglnSTI | 0.5498 |
| d1lagSTI | 0.0000 |
| d1laglnSTI | 0.0000 |
| GDPreal | 0.7966 |
| lnGDPreal | 0.6326 |
| d1GDPreal | 0.0000 |
| d1lnGDPreal | 0.0000 |
| Thailand | |
| lagSET | 0.7898 |
| laglnSET | 0.7470 |
| d1lagSET | 0.0000 |
| d1laglnSET | 0.0000 |
| GDPchain | 0.5216 |
| lnGDPchain | 0.4906 |
| d1GDPchain | 0.0000 |
| d1lnGDPchain | 0.0000 |

**METHODS FOR ROBUSTNESS CHECKS**

We ran three groups of multiple robustness checks to examine the stability of our results.

1. We modified data using original scale of variables. We used two method to compute GDP change due to NPI where we followed the initial steps in the main text and added these further steps:
2. The number was multiplied by the ratio of the nominal and real GDP to derive the inflation rate and multiplied by four, since GDP data is based quarterly. The resulting number is the absolute annual GDP change. To derive the percentage change, the absolute number was divided by the nominal GDP for 2019.
3. The resulting number to derive $\Delta GDP\left. \right|_{NPI}$ was divided by the average real GDP of the quarter and this was considered the percent change of GDP due to the NPI. To derive the impact in absolute terms, the GDP change rate was multiplied to the 2019 nominal GDP.
4. We considered the day of the week effects on H2 by adding dummy variables for the day.
5. We used a different weekly news trend using ‘coronavirus vaccine’ term from Google trends as a different gauge for public sentiment.

**RESULTS FOR ROBUSTNESS CHECKS**

**1. Using the original scale values**

We present coefficient values for H1 model, H2 model and GDP reduction for border closure and social distancing in Tables S3 to S5 respectively. We find that our conclusion is consistent when estimating the magnitude of decline of GDP due to effects of NPI, even when using the original scale of the variables.

Table S3. Resulting coefficients for H1 model, using original scale (A data-driven analysis of the economic cost of Non-Pharmaceutical Interventions: A cross-country comparison of Kenya, Singapore and Thailand; Kenya, Singapore and Thailand; 2022)

|  | Kenya | Singapore | Thailand |
| --- | --- | --- | --- |
| Original scale | 683.0 | 2.836 | 276* |
|  | (-69.67 to 1435.7) | (-1.899 to 7.571) | (96.50 to 455.5) |
| adj. R^2^ | 0.783 | 0.272 | 0.708 |

Note: *means significant values with p value < 0.05, 95% CIs in parenthesis

Table S4. Resulting coefficients for H2 model, using original scale (A data-driven analysis of the economic cost of Non-Pharmaceutical Interventions: A cross-country comparison of Kenya, Singapore and Thailand; Kenya, Singapore and Thailand; 2022)

|  | **AR (0)** | **AR (1)** | **AR (2)** | **AR (1) MA (1)** | **AR (1) MA (2)** |
| --- | --- | --- | --- | --- | --- |
| **Border closure** | | | | | |
| Kenya | -269.3* | -23.82 | -13.37 | -19.71 | -27.01 |
|  | (-354.2 to -184.4) | (-75.97 to 28.33) | (-13.37 to -13.37) | (-74.04 to 34.62) | (-88.07 to 34.05) |
| Singapore | -63.22* | -56.27 | -9.267 | -21.78 | -9.806 |
|  | (-106.8 to -19.68) | (-161.2 to 48.68) | (-56.17 to 37.63) | (-73.45 to 29.89) | (-65.60 to 45.98) |
| Thailand | -98.57* | -40.82* | -36.88* | -34.69 | -39.11* |
|  | (-144.6 to -52.52) | (-73.86 to -7.778) | (-73.15 to -0.599) | (-72.37 to 2.984) | (-69.95 to -8.270) |
| **Social distancing** | | | | | |
| Kenya | -41.82* | -12.8* | -6.576 | -6.246 | -6.463 |
|  | (-74.43 to -9.207) | (-16.53 to -9.079) | (-6.576 to -6.576) | (-26.44 to 13.95) | (-26.16 to 13.24) |
| Singapore | -255.2* | -287.2* | -163* | -180.7* | -161* |
|  | (-298.9 to -211.6) | (-446.1 to -128.2) | (-259.6 to -66.29) | (-293.1 to -68.37) | (-264.6 to -57.48) |
| Thailand | -55.97 | -22.19 | -18.23 | -19.87 | -21.13 |
|  | (-148.3 to 36.38) | (-44.69 to 0.298) | (-49.01 to 12.55) | (-49.30 to 9.549) | (-53.55 to 11.28) |

Note: *means significant values with p value < 0.05, 95% CIs in parenthesis

We observe a slight difference in results when original scale results are compared with log-scale results (Table S5 versus Table 2). In the main text, we present the log-scale results since this corresponds directly to percentage change, which is the usual way to describe the change in GDP.

For Kenya, we observe a wider range of GDP decline when using the original scale compared with log-scale. Meanwhile, the results from Singapore and Thailand were less affected by the choice of scale. An explanation could be that the stock market in Kenya is less well developed hence the results are less robust. From a policy-making perspective, we need to acknowledge and factor in these uncertainties in the decision making. As a caution, the countries’ context needs to be assessed to understand the appropriateness of applying the method and its implication on policy decisions.

Table S5. Estimated GDP reduction for border closure and social distancing using original scale, in percentage (A data-driven analysis of the economic cost of Non-Pharmaceutical Interventions: A cross-country comparison of Kenya, Singapore and Thailand; Kenya, Singapore and Thailand; 2022)

| **Country** | **Border closure** | | | **Social distancing** | | |
| --- | --- | --- | --- | --- | --- | --- |
|  | Best fit | Average | Range | Best fit | Average | Range |
| ***Method 2*** | | | | | | |
| Kenya | -0.37 | -1.92 | -0.37 to -7.55 | -0.18 | -0.38 | -0.18 to -1.17 |
| Singapore | -0.02 | -0.05 | -0.02 to -0.13 | -0.34 | -0.40 | -0.34 to -0.60 |
| Thailand | -0.36 | -0.48 | -0.34 to -0.97 | -0.20 | -0.26 | -0.18 to -0.55 |
| ***Method 3*** | | | | | | |
| Kenya | -0.37 | -1.90 | -0.37 to -7.48 | -0.18 | -0.37 | -0.17 to -1.16 |
| Singapore | -0.02 | -0.06 | -0.02 to -0.15 | -0.40 | -0.46 | -0.39 to -0.70 |
| Thailand | -0.47 | -0.65 | -0.47 to -1.33 | -0.27 | -0.35 | -0.25 to -0.75 |

**2. Exploring day of the week effect on H2**

We present coefficient values for the H2 model for border closure and social distancing using log-scale of the variables in Tables S6. There is a general pattern of decreasing stock market index when the NPIs were announced, following the results of the main analysis.

Table S6. Resulting coefficients for H2 model with day of the week (log-scale) (A data-driven analysis of the economic cost of Non-Pharmaceutical Interventions: A cross-country comparison of Kenya, Singapore and Thailand; Kenya, Singapore and Thailand; 2022)

|  | **AR (0)** | **AR (1)** | | **AR (2)** | | **AR (1) MA (1)** | **AR (1) MA (2)** | | |
| --- | --- | --- | --- | --- | --- | --- | --- | --- | --- |
| **Border closure** | | | | | | | | | |
| Kenya | -0.117* | | -0.0138 | | -0.0102 | -0.0126 | | -0.0148 |  |
|  | (-0.154 to -0.0796) | | (-0.0407 to 0.0132) | | (-0.0386 to 0.0182) | (-0.0411 to 0.0159) | | (-0.0441 to 0.0145) |  |
| Singapore | -0.0174* | | -0.0171 | | -0.00374 | -0.00699 | | -0.00353 |  |
|  | (-0.0343 to -0.0005) | | (-0.0567 to 0.0226) | | (-0.0208 to 0.0133) | (-0.0265 to 0.0125) | | (-0.0248 to 0.0177) |  |
| Thailand | -0.0658* | | -0.0280 | | -0.0247 | -0.0232 | | -0.0256 |  |
|  | (-0.0992 to -0.0325) | | (-0.0667 to 0.0107) | | (-0.0505 to 0.00109) | (-0.0489 to 0.00256) | | (-0.0513 to 0.00001) |  |
| **Social distancing** | | | | | | | | | |
| Kenya | -0.0224* | | -0.00934* | | -0.00616 | -0.00612 | | -0.00633 |  |
|  | (-0.0387 to -0.0060) | | (-0.0125 to -0.0062) | | (-0.0155 to 0.0032) | (-0.0164 to 0.0042) | | (-0.0162 to 0.0036) |  |
| Singapore | -0.0907* | | -0.0942* | | -0.0612* | -0.068* | | -0.059* |  |
|  | (-0.107 to -0.0749) | | (-0.166 to -0.0225) | | (-0.107 to -0.0156) | (-0.122 to -0.0142) | | (-0.109 to -0.0090) |  |
| Thailand | -0.0526 | | -0.0179 | | -0.0155 | -0.0172 | | -0.0173 |  |
|  | (-0.126 to 0.0210) | | (-0.0381 to 0.0022) | | (-0.0445 to 0.0134) | (-0.0466 to 0.0122) | | (-0.0453 to 0.0107) |  |

Note: *means significant values with p value < 0.05, 95% CIs in parenthesis

We observe that the results are similar for all the specifications when Table S6 is compared with Table 1; Table S7 with Table 2. Our results are robust when day-of-week fixed effect is controlled. In general, since our method resembles a before-and-after comparison, our results will be robust when adding in fixed effects.

Table S7. Estimated GDP reduction for border closure and social distancing using day of the week effect (log-scale), in percentage (A data-driven analysis of the economic cost of Non-Pharmaceutical Interventions: A cross-country comparison of Kenya, Singapore and Thailand; Kenya, Singapore and Thailand; 2022)

|  | Border closure | | | Social distancing | | |
| --- | --- | --- | --- | --- | --- | --- |
| Country | Best fit | Average | Range | Best fit | Average | Range |
| Kenya | -0.07 | -0.22 | -0.07 to -0.77 | -0.04 | -0.06 | -0.04 to -0.15 |
| Singapore | -0.05 | -0.09 | -0.04 to -0.22 | -0.77 | -0.86 | -0.74 to -1.19 |
| Thailand | -0.21 | -0.27 | -0.19 to -0.55 | -0.143 | -0.20 | -0.13 to -0.44 |

Note: Best fit are not the same across three countries. Both the average and range (highest to lowest results) were derived from the five models

**3. Using ‘coronavirus vaccine’ news trend instead of ‘coronavirus’**

We present coefficient values for the H2 model for border closure and social distancing using log-scale of the variables in Tables S8. There is a general pattern of decreasing stock market index when the NPIs were announced, except for border closure in Kenya. This might be the case since the stock market index in Kenya is less well developed hence the results are less robust.

We observe that results in Thailand were not affected when results using ‘coronavirus vaccine’ trend is compared with results using ‘coronavirus’ trend (Table S8 versus Table 1). For Singapore, the results for social distancing were not affected. Results for border closure changed slightly with higher magnitudes, and all results became significant at 5%. One implication is that ‘coronavirus vaccine’ may be a better indicator for public sentiment and panic towards the pandemic in Singapore. But, the overall ranges of cost of border closure between using ‘coronavirus’ trend and ‘coronavirus vaccine’ trend overlapped for Singapore (Table S9 versus Table 2). For Kenya, the average of the five models resulted in a positive GDP for border closure. An explanation could be that the stock market in Kenya is less well developed hence the results are less robust, which was observed in the other robust analysis. Furthermore, there were lots of misinformation for COVID-19 vaccine during the pandemic. Hence, we chose to control ‘coronavirus’ trend in the baseline results.

Table S8. Resulting coefficients for H2 model using ‘coronavirus vaccine’ news trend (log-scale) (A data-driven analysis of the economic cost of Non-Pharmaceutical Interventions: A cross-country comparison of Kenya, Singapore and Thailand; Kenya, Singapore and Thailand; 2021)

|  | **AR (0)** | **AR (1)** | | **AR (2)** | | **AR (1) MA (1)** | **AR (1) MA (2)** | | |
| --- | --- | --- | --- | --- | --- | --- | --- | --- | --- |
| **Border closure** | | | | | | | | | |
| Kenya | -0.0632 | | 0.0139* | | 0.0217* | 0.02* | | 0.0208* |  |
|  | (-0.0956 to -0.0308) | | (0.0047 to 0.0232) | | (0.0103 to 0.0330) | (0.0051 to 0.0349) | | (0.0052 to 0.0365) |  |
| Singapore | -0.00835* | | -0.0264* | | -0.015* | -0.0162* | | -0.0155* |  |
|  | (-0.0161 to -0.0006) | | (-0.0522 to -0.0006) | | (-0.0293 to -0.0008) | (-0.0296 to -0.0028) | | (-0.0298 to -0.0012) |  |
| Thailand | -0.0654* | | -0.0322 | | -0.0257 | -0.0235 | | -0.0276 |  |
|  | (-0.1040 to -0.0267) | | (-0.0751 to 0.0107) | | (-0.0541 to 0.0027) | (-0.0530 to 0.0061) | | (-0.0575 to 0.0023) |  |
| **Social distancing** | | | | | | | | | |
| Kenya | -0.0335* | | -0.0126* | | -0.00653 | -0.00764 | | -0.00707 |  |
|  | (-0.0523 to -0.0148) | | (-0.0241 to -0.0010) | | (-0.0145 to 0.0014) | (-0.0159 to 0.0007) | | (-0.0149 to 0.0007) |  |
| Singapore | -0.0956* | | -0.108* | | -0.0683* | -0.0751* | | -0.0698* |  |
|  | (-0.1120 to -0.0790) | | (-0.1570 to -0.0583) | | (-0.1120 to -0.0241) | (-0.1250 to -0.0250) | | (-0.1170 to -0.0227) |  |
| Thailand | -0.0614 | | -0.0205 | | -0.0184 | -0.0201 | | -0.0201 |  |
|  | (-0.1310 to 0.0084) | | (-0.0383 to -0.0026) | | (-0.0440 to 0.0072) | (-0.0451 to 0.0049) | | (-0.0462 to 0.0060) |  |

Note: *means significant values with p value < 0.05, 95% CIs in parenthesis

Table S9. Estimated GDP reduction for border closure and social distancing using vaccine trend, in percentage (A data-driven analysis of the economic cost of Non-Pharmaceutical Interventions: A cross-country comparison of Kenya, Singapore and Thailand; Kenya, Singapore and Thailand; 2021)

|  | Border closure | | | Social distancing | | |
| --- | --- | --- | --- | --- | --- | --- |
| Country | Best fit | Average | Range | Best fit | Average | Range |
| Kenya | 0.14 | 0.03 | 0.14 to -0.42 | -0.04 | -0.08 | -0.04 to -0.22 |
| Singapore | -0.19 | -0.21 | -0.11 to -0.33 | -0.86 | -0.98 | -0.86 to -1.36 |
| Thailand | -0.21 | -0.28 | -0.20 to -0.54 | -0.167 | -0.23 | -0.15 to -0.51 |

Note: Best fit are not the same across three countries. Both the average and range (highest to lowest results) were derived from the five models
